# Potential Role of Selected miRNAs in the Pathogenesis of Autoimmune Thyroid Diseases in Children and Adolescents

**DOI:** 10.3390/biomedicines12040731

**Published:** 2024-03-25

**Authors:** Beata Sawicka, Anetta Sulewska, Agnieszka Kulczyńska-Przybik, Filip Bossowski, Maciej Dulewicz, Hanna Borysewicz-Sańczyk, Barbara Mroczko, Jacek Nikliński, Artur Bossowski

**Affiliations:** 1 Department of Pediatrics, Endocrinology, Diabetology, with Cardiology Divisions, Medical University of Bialystok, 15-089 Bialystok, Poland; 2 Department of Clinical Molecular Biology, Medical University of Bialystok, 15-089 Bialystok, Poland; anetta.sulewska@umb.edu.pl (A.S.);; 3 Department of Neurodegeneration Diagnostics, Medical University of Bialystok, 15-089 Bialystok, Polandbarbara.mroczko@umb.edu.pl (B.M.)

**Keywords:** miRNA, Hashimoto’s thyroiditis, Graves’ disease, thyroid nodular disease

## Abstract

Background: Many epigenetic factors, including microRNAs, are involved in the process of changing gene expressions. Small non-coding RNA molecules, called miRNAs, are responsible for regulating gene translation by silencing or degrading target mRNAs. It is acknowledged that for many diseases, they may be novel diagnostic and prognostic biomarkers. Patients with autoimmune thyroid diseases are more likely to develop nodules in the thyroid tissue, and Hashimoto’s thyroiditis and Graves’ disease predispose patients to thyroid cancer. We evaluated the concentrations of microRNA molecules (miR-15a-5p, miR-126-3p, miR-142-5p, miR-21-5p, miR-150-5p) in the blood of children with thyroid disorders. In addition, we wished to identify molecules whose change in concentration predisposes to the development of thyroid cancer. Aim: The aim of this study is to evaluate selected epigenetic elements by analyzing the levels of miR-15a-5p, miR-126-3p, miR-142-5p, miR-150-5p and miR-21-5p in the blood of pediatric patients with Graves’ disease (*n* = 25), Hashimoto’s thyroiditis (*n* = 26) and thyroid nodular disease (*n* = 20) compared to a control group of healthy children (*n* = 17). Materials and Methods: The study consists of groups of children and adolescents aged 10–18 years with autoimmune thyroid disease, with thyroid nodular disease compared to a control group. The miR-15a-5p, miR-126-3p, miR-142-5p, miR-21-5p and miR-150-5p molecules were determined through an immunoenzymatic assay using BioVendor reagents. Results: There is a statistically significant decrease in the expression of the miR-15a-5p in children with Graves’ disease (21.61 vs. 50.22 amol/μL, *p* = 0.03) and in patients with thyroid nodular disease compared to controls (20.23 vs. 50.22 amol/μL, *p* = 0.04). Higher levels of the miR-142-5p molecule are found in patients with thyroid disease (with GD-3.8 vs. 3.14 amol/μL, *p* = 0.01; with HT-3.7 vs. 3.14 amol/μL, *p* = NS, with thyroid nodular disease-4.16 vs. 3.14 amol/μL, *p* = 0.04). Lower levels of miR-126-3p were noted in the GD group compared to the control group (7.09 vs. 7.24 amol/μL, *p* = 0.02). No statistically significant changes in the expressions of miR-150-5p and miR-21-5p molecules were observed in the study groups. Conclusions: 1. The overexpression of the miR-142-5p molecule occurs in children and adolescents with thyroid diseases. 2. Decreased blood levels of miR-15a-5p predispose patients to the formation of focal lesions in the thyroid gland. 3. Identifying a lower expression of the miR-126-3p molecule in the blood of children with GD requires careful follow-up for the development of focal lesions in the thyroid gland and evaluation for their potential malignancy.

## 1. Introduction

Among the multifactorial pathogenesis of autoimmune thyroid diseases (AITDs) are genetic predisposition and environmental factors. Graves’ disease (GD) and Hashimoto’s thyroiditis (HT) are associated with a dysfunction in the immune system. The impaired immune tolerance of thyroid antigen molecules results in a defective immune response. There is an incorrect identification of the body’s own cells, which impairs the safety of tissues and organs. Abnormal immunotolerance results from impaired suppression involving regulatory T cells in peripheral immunocompetent tissues (Tregs) [1]. It is known that patients with autoimmune thyroid diseases are more likely to develop focal lesions in the thyroid tissue, and Hashimoto’s thyroiditis and Graves’ disease predispose patients to the development of thyroid cancer [2,3,4,5]. To date, many mutations in oncogenes and tumor suppressor genes have been found in patients with malignant tumors. It is known that most of the human genome does not have the ability to encode proteins. Only 2% have this capability. RNA encodes most of the non-coding genome. Mutations in RNA can cause many human diseases, including autoimmunity, or cancer [6].

MicroRNAs (miRNAs) are small 22-nucleotide non-coding molecule RNAs that regulate gene translation by silencing or degrading target mRNAs. By binding to the 3′ non-translational regions (3′ UTR) of the RNAs of protein-coding genes, they signal the translation or degradation of the messenger molecule. MiRNAs are involved in many biological processes, such as proliferation, metabolism, hemostasis, apoptosis and inflammation, the dysfunction of which may play an important role in the pathogenesis of many diseases. Thus, they may be considered as new diagnostic and prognostic biomarkers for a variety of defects.

Studies have shown that miR-15a affects pancreatic β-cells, thereby playing a role in insulin synthesis. Increased levels of miR-15a are observed in patients with diabetes. The more metabolically unstable the diabetes, the higher the amount in serum [7]. Downregulated levels of miR-15a are observed in many cancers, including colorectal cancer [8], liver cancer [9], prostate cancer [10], chronic lymphocytic leukaemia [11], nasopharyngeal carcinoma [12], malignant melanoma [13], human brain glioma [14] and breast cancer [15].

In contrast, another miRNA that regulates the expressions of many cancer-related genes is miR-126-3p. It is also endothelium-specific because it also regulates angiogenesis and blood vessel integrity [16]. It acts either as a tumor suppressor or as an oncogene in various types of cancer. As has been reported in published studies, the miR-126-3p expression is found to be downregulated in malignant thyroid cancer samples compared to benign thyroid nodules. A decreased miR-126-3p expression is associated with clinically more aggressive papillary thyroid cancer [17].

The miR-142-5p molecule also presents high specificity for hematopoietic cells and increased levels are observed in many pathological conditions such as inflammation [18], immune disorders [19] and cancers, for example, retinoblastoma [20] and breast cancer [21]. Moreover, the downregulation of miR-142-5p may serve as an additional indicator of metastasis in thyroid cancer [22].

The miR-21-5p is a typical onco-miRNA. It is one of the first identified miRNA molecules. It modulates the expressions of many tumor-related target genes. In addition to tissues, it is found in various types of extracellular fluids, such as plasma, serum, cerebrospinal fluid, saliva, gastric fluid, pancreatic juice, sputum and pancreatic cyst fluid. A high level of the miR-21-5p expression is a negative predictor of survival in various cancers [23,24,25]. It plays an important role in vascular smooth muscle cell proliferation and apoptosis, cardiac cell growth and death, aortic valve calcification and cardiac fibroblast function [26]. Moreover, the expression of miR-21-5p is significantly upregulated in patients with Hashimoto’s thyroiditis [27].

miR-150-5p is another microRNA with an expression that is an important regulator of immune cell differentiation and activation. A particular selective expression of miR-150-5p occurs from lymph nodes, the spleen and in mature B and T lymphocytes. The finding of lower levels of miR-150-5p in cancer patients predicted worse responses to chemotherapy and shorter survival. In contrast, a high expression of miR-150 in patient tumor samples indicates a better prognosis and better response to chemotherapy. Moreover, the overexpression of miR-150-5p leads to a reduced migration and invasion of colorectal cancer cells. MiR-150-5p also acts as a suppressor of metastasis in colorectal cancer [28].

There are very few studies evaluating the concentrations of these molecules in children. We would like to assess the concentrations of microRNA molecules (miR-15a-5p, miR-126-3p, miR-142-5p, miR-21-5p, miR-150-5p) in children with thyroid disorders, the predisposition to the development of focal lesions in thyroid gland and the risk of developing malignancy, considering predisposition to cancer [29,30].

## 2. Materials and Methods

This study was performed with 25 patients with newly diagnosed GD (mean age 14.36 ± 3.6), 26 with newly diagnosed HT (mean age, 14.18 ± 3.9), 20 with a nodular goiter in euthyroid (mean age, 13.7 ± 2.9) and 17 children without any autoimmune diseases (mean age 12.58 ± 2.6) recruited from the Department of Pediatrics, Endocrinology, Diabetology, with Cardiology Divisions and from the Pediatric Endocrinology Outpatient Clinic of Medical University in Bialystok. The diagnoses of autoimmune thyroid diseases were based on medical history, physical examination, laboratory and ultrasound investigations [31,32]. Patients with thyroid diseases had no other autoimmune comorbidities. We excluded other endocrinopathies. A clinical diagnosis of hyperthyroidism in GD was confirmed by elevated thyroid hormones in serum and the suppression of TSH (thyroid- stimulating hormone) to values close to zero with positive antibodies against the receptor for thyroid-stimulating hormone (TRAb = anti-TSH), positive anti-thyroid peroxidase antibodies (aTPO) and anti-thyroglobulin antibodies (aTG). In clinically evident hypothyroidism in HT, serum TSH levels were elevated at reduced concentrations of thyroid hormones (fT4—free thyroxine, fT3—triiodothyronine), usually accompanied by elevated thyroid antibodies (aTPO and/or aTG, rarely blocking anti-TSH). Children and adolescents with GD and HT exhibited a typical thyroid picture for AITD on ultrasound. Patients with nodular goiters underwent fine-needle aspiration biopsies (FNABs) to exclude thyroid cancer. All patients started appropriate therapy for autoimmune thyroid pathology. Patients with newly diagnosed GD started therapy with methimazole and b-blockers, administered orally. Patients with newly diagnosed HT received l-thyroxine orally in appropriate doses as part of their therapy. The control group consisted of 17 healthy children with no personal or family history of any AITDs. They were euthyroid and negative for thyroid antibodies. All controls had normal thyroid glands on ultrasonography. Before enrollment, all patients’ and controls’ parents and all children over 16 years old signed informed consent forms. The protocol for the study was approved by the Local Bioethical Committee at the Medical University of Bialystok.

### 2.1. Assessment of the Thyroid Hormone Concentration and Anti-Thyroid Antibody Titers

Blood for analysis was collected in the morning from the basilic vein. The serum levels of free thyroxine (fT4), free triiodothyronine (fT3) and TSH were determined based on electrochemiluminescence ‘ECLIA’ with a Cobas E411 analyzer (Roche Diagnostics, Rotkreuz, Switzerland). Normal values for fT4 ranged between 1.1 and 1.7 ng/dL; for fT3, between 2.3 and 5.0 pg/mL; and for TSH, between 0.28 and 4.3 (µIU/L). TR-Ab, aTPO and aTG antibodies were measured in all samples using ECLIA with a Modular Analytics E170 analyzer (Roche Diagnostics). The positive values for antithyroid antibodies were >1.75 U/L for TRAb, >34 IU/mL for aTPO-Abs and >115 IU/mL for aTG-Abs. The blood samples for microRNAs and for thyroid hormones and antithyroid antibodies analyses were harvested at the same time points.

### 2.2. Assessment of miR-15a-5p, miR-126-3p, miR-142-5p, miR-21-5p and miR-150-5p Levels

Whole blood for miRNA analysis was collected in PAX-Gene Blood RNA Tubes (IVD) (Qiagen, Germantown, MD, USA) and treated according to the manufacturer’s protocol. Blood for miRNA determination was provided before the drug treatment was started. Subsequently, the blood underwent centrifugation at 2000× *g* for 20 min at zero acceleration and deceleration within a refrigerated centrifuge. The resulting supernatant was transferred into 2 mL Eppendorf tubes. To obtain purified plasma, the supernatant was subjected to a secondary centrifugation step at 20,000× *g* for 15 min in a refrigerated centrifuge. Hemolysis in the plasma samples was assessed through visual examination and spectrophotometric analysis at a wavelength of 414 nm. The plasma was then divided into 0.5 mL aliquots and promptly stored at −80 °C until the subsequent RNA extraction process. The miRNA from the plasma was extracted using a miRNA Isolation Kit Serum/Plasma (BioVendor R&D, Brno, Czech Republic) according to the manufacturer’s protocol. The isolated miRNAs were quantified via hybridization to complementary biotinylated DNA oligonucleotide probes. The DNA/RNA hybrids were then captured using a microtiter plate-immobilized monoclonal antibody specific to perfectly match the DNA/miRNA hybrids using miREIA—a novel, immunoassay-based method of miRNA quantification—following the manufacturer’s instructions (BioVendor R&D, Brno, Czech Republic). The next steps followed standard ELISA protocols. The results were reported as concentrations of miR-15a-5p (amol/μL), miR-126-3p (amol/μL), miR-142-5p (amol/μL), miR-21-5p (amol/μL) and miR-150-5p (amol/μL) in the samples.

### 2.3. Statistical Analysis

The *p*-value obtained using Fisher’s exact test [33] was used to assess the association between miRNA prevalence and the median unbiased estimator (mid-*p*) of the odds ratio (as well as its 95% exact confidence interval). Parametric or non-parametric methods were used to determine statistically significant differences between disease-defined groups, depending on whether the assumptions of normality and homogeneity of variance were met. Due to the issue of multiple tests during the post hoc analysis, the *p*-value correction method of the false discovery rate was used [34,35]. A *p*-value < 0.05 was considered significant for all calculations. The R software (version 4.2.3.) environment was used for all calculations [36].

## 3. Results

Age and anthropometric parameters in the study groups of children with GD, HT and nodular goiter compared to the control group did not have statistically significant differences (Table 1).

### 3.1. Results for miR-15a-5p

**Figure 1:**
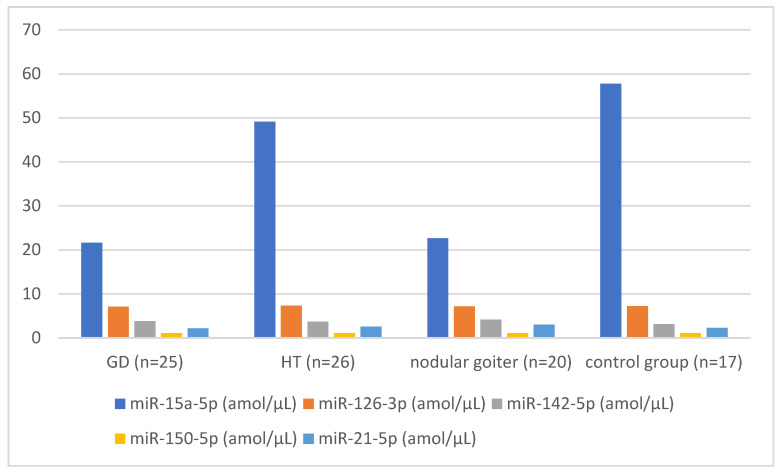
Management of particular miRNA molecules (miR-15a-5p, miR-126-3p, miR-142-5p, miR-21-5p and miR-150-5p) in groups with Graves’ disease (GD), with Hashimoto’s thyroiditis (HT) and with nodular goiter and in control group.

Our study shows that miR-15a-5p levels were lower in patients with GD and with nodular goiter compared to those in healthy children (21.61 amol/µL in GD vs. 50.2 amol/µL in control group, *p* = 0.03; 20.3 amol/µL in nodular goiter vs. 50.22 amol/µL in control group, *p* = 0.049, Figure 1). There were lower levels in patients with HT compared to the control group, but not statistically significant (42.99 amol/µL vs. 50.22 amol/µL; *p* = NS) (Table 2). In comparing both groups with autoimmune diseases, HT and GD, miRNA-15a-5p was higher in patients with HT (49.15 amol/µL vs. 21.61 amol/µL; *p* = NS) (Table 3). A negative correlation between aTPO levels and miRNA 15-a-5p levels was identified in the group of patients with GD. Moreover, a positive correlation between miRNA15a-5p levels and TRAb titers was observed in GD patients (Table 4 and Table 5).

### 3.2. Results for miR-126-3p

**Figure 2:**
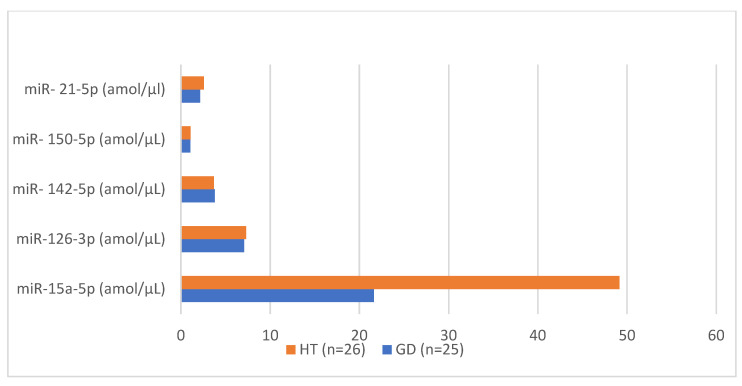
Management of particular miRNA molecules (miR-15a-5p, miR-126-3p, miR-142-5p, miR-21-5p and miR-150-5p) in group with Graves’ disease (GD) compared to group with Hashimoto’s thyroiditis (HT).

miR-126-3p levels were lower in patients with GD compared to healthy children (7.09 amol/µL in GD group vs. 7.24 amol/µL in control group, *p* = 0.02). There were higher levels in patients with HT compared to control group, but not statistically significant (7.31 amol/µL vs. 7.24 amol/µL; *p* = NS). In children with nodular goiter, miRNA-126-3p levels were slightly lower than in the control group (7.14 amol/µL vs. 7.24 amol/µL; *p* = NS) (Table 2, Figure 1). In comparing both groups with autoimmune diseases, HT and GD, miRNA-126-3p was higher in patients with HT (7.31 amol/µL vs. 7.09 amol/µL; *p* = NS) (Table 3, Figure 2). A positive correlation between aTPO levels and miRNA-126-3p was found in the group of patients with HT (r = 0.478; *p* < 0.05; Table 5). A negative correlation between fT3 levels and miRNA 126-3p levels was identified in the group of patients with nodular goiter (Table 6).

### 3.3. Results for miR-142-5p

Our study shows that miR-142-5p levels were significance higher in patients with GD and with nodular goiter compared to those in healthy children (3.8 amol/µL in GD vs. 3.14 amol/µL in control group, *p* = 0.01; 4.16 amol/µL in nodular goiter vs. 3.14 amol/µL in control group, *p* = 0.04, Figure 1). Also, there were higher levels in patients with HT compared to the control group, but not statistically significant (3.7 amol/µL vs. 3.14 amol/µL; *p* = NS) (Table 2). In comparing both groups with autoimmune diseases, HT and GD, miR-142-5p levels were similar in both patient groups (3.7 amol/µL in HT vs. 3.8 amol/µL in GD; *p* = NS) (Table 3, Figure 2). A positive correlation between aTPO levels and miRNA-142-5p was identified in the group of patients with HT (r = 0.491; *p* = 0.917; Table 5). There was no statistically significant correlation between miR-142-5p, thyroid hormones levels and anti-thyroid antibodies in the other studied groups (Table 4 and Table 6).

### 3.4. Results for miR-150-5p

miR-150-5p levels were similar in patients with GD, HT and nodular goiter compared to those in healthy children (1.07 amol/µL in GD vs. 1.06 amol/µL in control group, *p* = NS; 1.08 amol/µL in HT vs. 1.06 amol/µL in control group, *p* = NS; 1.07 amol/µL in nodular goiter vs. 1.06 amol/µL in control group, *p* = NS) (Table 2, Figure 1). In comparing both groups with autoimmune diseases, HT and GD, miR-150-5p levels were similar in both patient groups (1.06 amol/µL in HT vs. 1.07 amol/µL in GD; *p* = NS) (Table 3, Figure 2). A positive correlation between aTPO levels and miRNA-150-5p was identified in the group of patients with HT (r = 0.485; *p* = 0.019; Table 5). There was no statistically significant correlation between miRNA-150-5p thyroid hormones levels and anti-thyroid antibodies in the groups of patients with GD and with nodular goiter (Table 4 and Table 6). In the group of patients with GD, a positive correlation was found between miR-150-5p and miR-21-5p (r = 0.495; Table 7), and also between miR-150-5p and miRNA-142-5p (r = 0.486; Table 7).

### 3.5. Results for miR-21-5p

Our study shows that miR-21-5p levels were not significance higher in patients with HT or with nodular goiter compared to those in healthy children (2.58 amol/µL in HT vs. 2.28 amol/µL in control group, *p* = NS; 3.04 amol/µL in nodular goiter vs. 2.28 amol/µL in control group, *p* = NS). MiR-21-5p levels were lower in patients with GD compared to healthy children (2.16 amol/µL in GD vs. 2.28 amol/µL in control group, *p* = NS) (Table 2, Figure 1). In comparing both groups with autoimmune diseases, HT and GD, miR-21-5p levels were higher in the group with HT than the group with GD (2.58 amol/µL in HT vs. 2.16 amol/µL in GD; *p* = NS) (Table 3). A positive correlation between aTPO levels and miRNA-21-5p was identified in the group of patients with HT (r = 0.489; *p* = 0.018; Table 5). There was no statistically significant correlation between miR-21-5p thyroid hormones levels and anti-thyroid antibodies in the patients with GD or nodular goiter (Table 4 and Table 6). In the group of patients with nodular goiter, a positive correlation was found between miR-21-5p and miR-150-5p (r = 0.555; Table 8), and also between miR-21-5p and miR-142-5p (r = 0.487; Table 8).

## 4. Discussion

The different levels of miRNAs were associated with GD and HT, which may play a role in the pathogenesis of these diseases. It is known that autoimmune thyroid diseases predispose patients to the development of thyroid cancer. In recent years, several reports have been published confirming different serum or tissue miRNA levels in autoimmune thyroid diseases and thyroid cancers in adults. There are still no reports on children evaluating miRNA levels as a risk for autoimmune endocrine disorders.

In the present study, none of the observed miRNAs were differentially expressed in the sera of HT patients compared to healthy controls. Our study showed that miR-15a-5p levels in serum were lower in GD patients and with nodular goiter compared to healthy children. Moreover, miR-142-5p levels were higher in the same groups. Only miR-126-3p serum levels were lower in patients with GD.

As a regulator of the expression of many cancer-related genes, downregulated miR-126-3p may impact the development of malignant thyroid cancer. Xiong Y. et al. [17] reported that a reduced miR-126-3p expression is associated with papillary thyroid cancer. The risk of developing thyroid cancer in nodules in patients with autoimmune diseases is higher than in patients with nodular goiter without autoimmune diseases. So, GD patients with lower levels of miR-126-3p require careful follow-up for the development of focal lesions in the thyroid gland. This still requires further studies. There are no studies in the literature evaluating the molecule in the sera of GD patients.

In our study, we noticed that miR-142-5p levels were higher in patients with AITDs and with nodular goiter compared to healthy children, but not statistically significant higher levels in patients with HT compared to the control group. Trummer O. et al. showed significantly higher expression levels for miR-142-3p in patients with HT [27]. Zhu et al. reported positive associations of TgAb and miR-142-5p levels in HT serum [37]. In our subgroup analysis, we found no evidence of a significant correlation between antithyroid antibodies and miR-142-5p. It is possible that the detectable overexpression of miR-142-5p may be due to both active secretion as a result of inflammation in patients with AITD and the origin of autoimmune cell death or the proliferation of abnormal cells. We suspect that miR-142-5p may be a useful marker of abnormal thyrocytes.

In addition, Yao et al. found that miR-21-5p may be related to atherosclerosis in patients with subclinical hypothyroidism, and miR-150-5p seems to be a sensitive risk marker in predicting endothelial dysfunction in patients with arteriosclerosis [38]. We did not report any significant differences between miR-21-5p serum levels and thyroid hormones levels in the studied groups. To explore this topic further, it would be necessary to compare miRNA levels with lipid concentrations and other markers of atherosclerosis.

In reviewing the relevant literature, there are no studies evaluating these miRNAs in children’ serum compared to thyroid tissue. Jin et al. found that miR-15a was down-expressed in human papillary thyroid cancer (PTC) tissues [39]. They showed that the upregulation of miR-15a mimics could inhibit tumor growth. Hu J. et al. found that the miR-15a expression in the PTC tissues was lower compared with in nodular goiter tissues and perineoplastic thyroid tissues [40], so it might be a useful biomarker and promising target in the diagnosis of papillary thyroid carcinoma. The findings of Jiang et al. [41] and Zhang et al. [42] indicated that miR-15a and miR-21-5p may exert tumor-suppressive effects on PTC. On the other hand, Jianugo W. et al. demonstrated that the expression levels of these miRNAs were increased in PTC compared with those noted in normal tissues [43]. Further, Wu L. et al. confirmed that miR-21-5p was secreted by hypoxic thyroid cancer cells and transferred to endothelial cells and was upregulated in the sera of patients with PTC [44]. In our study, although miR-21-5p levels were not significantly higher in patients with HT or with nodular goiter and were lower in patients with GD compared to healthy children, in the future, it would be worthwhile to assess serum miR-21-5p levels in children with thyroid cancer.

The miRNAs can be differentially expressed in AITD patients in thyroid tissue, the orbit and/or serum or plasma. The ideal study would evaluate miRNAs in different tissues in the same patient. There are no studies in the literature evaluating serum miRNAs in children and adolescents with autoimmune thyroid diseases and nodular goiter. Despite the rather small number of study groups, we view these results as a prelude to further research. In the future, we plan to expand the size of the study group and add a group of thyroid cancer patients. To sum up, the analysis of different miRNAs in clinical practice could allow us to differentiate thyroid diseases, asses the risk of developing thyroid cancer and use appropriate treatments. These miRNAs can be useful in monitoring nodules in thyroid glands in patients with AITDs. The assessment of the serum levels of miRNAs may be a practical noninvasive method for the follow-up of patients’ therapy including thyroidectomy, but it still requires a lot of thorough research to improve our knowledge on the subject.

## 5. Conclusions

This study reveals the following conclusions:

1. The overexpression of the miR-142-5p molecule occurs in children and adolescents with thyroid diseases.
2. Decreased blood levels of miR-15a-5p predispose patients to the formation of focal lesions in the thyroid gland.
3. Identifying a lower expression of the miR-126-3p molecule in the blood of children with GD requires careful follow-up for the development of focal lesions in the thyroid gland and evaluation for their potential malignancy.

## Figures and Tables

**Table 1:** Clinical characteristics of patients with Graves’ disease (GD), with Hashimoto’s thyroiditis (HT) and with nodular goiter and of control group.

	GD (Mean ± SD)	*p*^	HT (Mean ± SD)	*p*^^	Nodular Goiter (Mean ± SD)	*p*^^^	Control Group
children (female/male)	25 (15/10)		26 (20/6)		20 (15/5)		17 (8/9)
age (years)	14.36 ± 3.6	NS	14.18 ± 3.9	NS	13.7 ± 2.9	NS	12.58 ± 2.6
fT4 (ng/dL)	**12.68 ± 33.5**	***p* < 0.001**	**1.18 ± 0.24**	***p* < 0.01**	2.31 ± 4.2	NS	1.55 ± 0.62
fT3 (pg/mL)	**17.99 ± 34.39**	***p* < 0.001**	2.54 ± 1.1	NS	4.93 ± 0.1	NS	3.61 ± 2.12
TSH (mIU/L)	**0.96 ± 0.22**	***p* < 0.001**	8.58 ± 20.45	NS	2.27 ± 1.54	NS	2.16 ± 0.95
anti-TSH (U/L)	**17.84 ± 31.86**						
aTG (IU/mL)	**248.3 ± 348.71**	***p* < 0.02**	**441.96 ± 442.89**	***p* < 0.001**	201.78 ± 433.6	NS	25.25 ± 22.95
aTPO (IU/mL)	**238.94 ± 182.76**	***p* < 0.001**	**172.31 ± 121.99**	***p* < 0.001**	43.34 ± 33.9	NS	23.92 ± 33.9
treatment	methamizole/ b-blocker		l-thyroxine				none

SD—Standard Deviation; *p*—*p*-value; NS—statistically non-significant—*p*-value > 0.05. *p*^—correlation between group of patients with GD compared to control group. *p*^^—correlation between group of patients with HT compared to control group. *p*^^^—correlation between group of patients with nodular goiter compared to control group.

**Table 2:** MiR-15a-5p, miR-126-3p, miR-142-5p, miR-21-5p and miR-150-5p in groups with Graves’ disease (GD), with Hashimoto’s thyroiditis (HT) and with nodular goiter compared to control group.

	Patients with GD (*n* = 25)	Patients with HT (*n* = 26)	Patients with Nodular Goiter (*n* = 20)	Control Group (*n* = 17)
miR-15a-5p amol/µL	**21.61 (*p* = 0.03) *p*^**	49.15 (*p* = NS) *p*^^	**22.63 (*p* = 0.04)** ***p*^^^**	57.74
miR-126-3p amol/µL	**7.09 (*p* = 0.02)** ***p*^**	7.31 (*p* = NS) *p*^^	7.14 (*p* = NS) *p*^^^	7.24
miR-142-5p amol/µL	**3.80 (*p* = 0.01)** ***p*^**	3.70 (*p* = NS) *p*^^	**4.16 (*p* = 0.04)** ***p*^^^**	3.14
miR-150-5p amol/µL	1.07 (*p* = NS) *p*^	1.08 (*p* = NS) *p*^^	1.07 (*p* = NS) *p*^^^	1.06
miR-21-5p amol/µL	2.16 (*p* = NS) *p*^	2.58 (*p* = NS) *p*^^	3.04 (*p* = NS) *p*^^^	2.28

*p*—*p*-value; NS—statistically non-significant—*p*-value > 0.05; *p*^—correlation between group of patients with GD compared to control group. *p*^^—correlation between group of patients with HT compared to control group. *p*^^^—correlation between group of patients with nodular goiter compared to control group.

**Table 3:** MiR-15a-5p, miR-126-3p, miR-142-5p, miR-21-5p and miR-150-5p in group with Graves’ disease (GD) compared to group with Hashimoto’s thyroiditis (HT).

	Patients with GD (*n* = 25)	Patients with HT (*n* = 26)
miR-15a-5p	21.61 amol/µL	49.15 amol/µL (*p* = NS)
miR-126-3p	7.09 amol/µL	7.31 amol/µL (*p* = NS)
miR-142-5p	3.8 amol/µL	3.7 amol/µL (*p* = NS)
miR-150-5p	1.07 amol/µL	1.08 amol/µL (*p* = NS)
miR-21-5p	2.16 amol/µL	2.58 amol/µL (*p* = NS)

*p*—*p*-value; NS—statistically non-significant—*p*-value > 0.05; *p*-correlation between group of patients with GD compared to group with HT.

**Table 4:** The correlation between miR-15a-5p, miR-126-3p, miR-142-5p, miR-21-5p and miR-150-5p levels and thyroid hormones levels and anti-thyroid antibodies in the group with Graves’ disease (GD).

	miR-150-5p	miR-21-5p	miR-15a-5p	miR-126-3p	miR-142-5p
TSH	r = −0.099 *p* = NS	r = −0.304 *p* = NS	r = −0.011 *p* = NS	r = 0.132 *p* = NS	r = −0.447 *p* = NS
fT4	r = 0.143 *p* = NS	r = 0.357 *p* = NS	r = 0.078 *p* = NS	r = 0.135 *p* = NS	r = 0.416 *p* = NS
fT3	r = 0.188 *p* = NS	r = 0.1343 *p* = NS	r = 0.109 *p* = NS	r = 0.119 *p* = NS	r = 0.293 *p* = NS
aTG	r = −0.342 *p* = NS	r = −0.555 *p* = NS	r = −0.445 *p* = NS	r = −0.422 *p* = NS	r = 0.7118 *p* = NS
aTPO	r = 0.429 *p* = NS	r = 0.081 *p* = NS	**r = −0.892** ***p* = 0.007**	r = −0.648 *p* = NS	r = −0.188 *p* = NS
TRAb	r = −0.37 *p* = NS	r = 0.015 *p* = NS	**r = 0.765** ***p* = 0.045**	r = 0.729 *p* = NS	r = 0.148 *p* = NS

*p*—*p*-value; NS—statistically non-significant—*p*-value > 0.05; r—correlation coefficient.

**Table 5:** The correlation between miR-15a-5p, miR-126-3p, miR-142-5p, miR-21-5p and miR-150-5p levels and thyroid hormones levels and anti-thyroid antibodies in the group with Hashimoto’ thyroiditis (HT).

	miR-150-5p	miR-21-5p	miR-15a-5p	miR-126-3p	miR-142-5p
TSH	r = −0.067 *p* = NS	r = −0.76 *p* = NS	r = −0.065 *p* = NS	r = 0.132 *p* = NS	r = −0.065 *p* = NS
fT4	r = 0.037 *p* = NS	r = 0.029 *p* = NS	r = 0.212 *p* = NS	r = 0.035 *p* = NS	r = 0.011 *p* = NS
aTG	r = 0.098 *p* = NS	r = 0.087 *p* = NS	r = 0.261 *p* = NS	r = 0.100 *p* = NS	r = 0.077 *p* = NS
aTPO	**r = 0.485** ***p* = 0.019**	**r = 0.489** ***p* = 0.018**	r = 0.152 *p* = NS	**r = 0.478** ***p* = 0.021**	**r = 0.491** ***p* = 0.017**

*p*—*p*-value; NS—statistically non-significant—*p*-value > 0.05; r—correlation coefficient.

**Table 6:** The correlation between miR-15a-5p, miR-126-3p, miR-142-5p, miR-21-5p and miR-150-5p levels and thyroid hormones levels and anti-thyroid antibodies in the group with nodular goiter.

	miR-150-5p	miR-21-5p	miR-15a-5p	miR-126-3p	miR-142-5p
TSH	r = 0.443 *p* = NS	r = 0.091 *p* = NS	r = −0.963 *p* = NS	r = 0.509 *p* = NS	r = 0.712 *p* = NS
fT4	r = 0.859 *p* = NS	r = −0.086 *p* = NS	r = −0.662 *p* = NS	r = 0.895 *p* = NS	r = 0.976 *p* = NS
fT3	r = −0.992 *p* = NS	r = −0.073 *p* = NS	r = 0.302 *p* = NS	**r = −0.999** ***p* = 0.029**	r = −0.977 *p* = NS
aTG	r = −0.568 *p* = NS	r = −0.212 *p* = NS	r = 0.914 *p* = NS	r = −0.628 *p* = NS	r = −0.806 *p* = NS
aTPO	r = 0.681 *p* = NS	r = −0.061 *p* = NS	**r = 0.592** ***p* = 0.007**	r = 0.624 *p* = NS	r = 0.403 *p* = NS

*p*—*p*-value; NS—statistically non-significant—*p*-value > 0.05; r—correlation coefficient.

**Table 7:** The correlations between miRNAs in the group with GD.

	miR-15a-5p	miR-150-5p	miR-21-5p	miR-126-3p	miR-142-5p
miR-15a-5p	r = 1	r = 0.175	r = −0.133	r = 0.018	r = −0.024
miR-150-5p	r = 0.175	r = 1	**r = 0.495**	r = −0.133	**r = 0.486**
miR-21-5p	r = −0.133	**r = 0.495**	r = 1	r = 0.28	**r = 0.533**
miR-126-3p	r = 0.018	r = −0.133	r = 20.8	r = 1	r = 0.3
miR-142-5p	r = −0.24	**r = 0.486**	**r = 0.533**	r = 0.03	r = 1

r—correlation coefficient.

**Table 8:** The correlations between miRNAs in the group with nodular goiter.

	miR-15a-5p	miR-150-5p	miR-21-5p	miR-126-3p	miR-142-5p
miR-15a-5p	r = 1	r = 0.247	r = −0.023	r = −0.269	r = 0.171
miR-150-5p	r = 0.247	r = 1	**r = 0.555**	r = −0.020	**r = 0.528**
miR-21-5p	r = −0.023	**r = 0.555**	r = 1	r = 0.043	**r = 0.487**
miR-126-3p	r = −0.269	r = −0.02	r = 0.043	r = 1	r = −0.075
miR-142-5p	r = 0.171	**r = 0.528**	**r = 0.487**	r = −0.075	r = 1

r—correlation coefficient

## Data Availability

Data are contained within the article.
